# Evolutionary conservation of *MLO* gene promoter signatures

**DOI:** 10.1186/s12870-019-1749-3

**Published:** 2019-04-17

**Authors:** Giuseppe Andolfo, Paolo Iovieno, Luigi Ricciardi, Concetta Lotti, Edgardo Filippone, Stefano Pavan, Maria Raffaella Ercolano

**Affiliations:** 1 Department of Agricultural Sciences, University of Naples “Federico II”, Via Università 100, 80055 Portici (Naples), Italy; 2 Department of Soil, Plant and Food Science, University of Bari “Aldo Moro”, Via Amendola 165/A, 70126 Bari, Italy; 3 Department of Agriculture, Food and Environmental Science, University of Foggia, via Napoli 25, 71100 Foggia, Italy; 4 Institute of Biomedical Technologies, National Research Council (CNR), Via Amendola 122/D, 70126 Bari, Italy

**Keywords:** Cis-acting regulatory element, Motif, *MLO*, Powdery mildew resistance, Transcription

## Abstract

**Background:**

Powdery mildew (PM) is a widespread fungal disease of plants in temperate climates, causing significant economic losses in agricultural settings. Specific homologs of the *MLO* gene family are PM susceptibility factors, as their loss-of function results in durable PM resistance (*mlo* resistance) in several plant species. The role of *MLO* susceptibility genes in plant-pathogen interactions is still elusive, however it is known that they are strongly upregulated following PM infection.

**Results:**

In this study, we investigated the structure of 414 Putative Promoter Regions (PPRs) of *MLO* genes and highlighted motif and regulatory element patterns related to genomic relationships among species and phylogenetic distance among homologs. A TC box-like motif and a thymine-rich motif were found to be overrepresented in *MLO* genes transcriptionally upregulated upon infection with PM fungi. As proof of concept, we showed that the expression of a melon (*Cucumis melo* L.) gene enriched for the motifs above mentioned was strongly upregulated upon infection with the PM fungus *Podosphaera xanthii*.

**Conclusion:**

While identifying a candidate *MLO* susceptibility gene in melon, this study provides insight on the transcriptional control of *MLO* genes and indicates diagnostic features useful to identify *MLO* susceptibility genes across species affected by the PM disease.

**Electronic supplementary material:**

The online version of this article (10.1186/s12870-019-1749-3) contains supplementary material, which is available to authorized users.

## Background

The powdery mildew (PM) disease, caused by ascomycete fungi from the order of Erysiphales, can severely affect the yield and the quality of several agricultural species cultivated in temperate regions, including melon (*Cucumis melo* L.). Chemical control of PM is generally costly and raises important environmental issues. Therefore, understanding molecular mechanisms underlying genotypic resistance to PM is of great interest for agricultural genetics and breeding.

Specific homologs of the *Mildew Locus O* (*MLO*) plant-specific gene family are PM susceptibility factors, as their loss-of-function results in a durable and broad-spectrum form of resistance, known as *mlo* resistance [1]. Originally discovered in barley, *mlo* resistance was later shown to occur in several other species, namely Arabidopsis, tomato, pea, pepper, tobacco and wheat [2–10], and proved to be effective against both epiphytic and endophytic PM species [10, 11]. Therefore, several authors suggested the opportunity of a plant breeding strategy based on the identification and inactivation of *MLO* susceptibility genes [1, 7].

With no exception known so far, *MLO* susceptibility genes belong to two phylogenetic clades, referred to as clade IV and V, which are specific for monocots and eudicots, respectively [12–16]. However, not all clade IV and clade V *MLO* genes are PM susceptibility factors. For example, while silencing of the tomato gene *SlMLO1* results in *mlo* resistance to the powdery mildew pathogen *Oidium neolycopersici*, silencing of the other clade V genes *SlMLO3*, *SlMLO5* and *SlMLO8* is still associated with a susceptible phenotype [17]. Previous investigations showed *MLO* homologs playing a major role in PM susceptibility, including barley *HvMLO*, tomato *SlMLO1* and pepper *CaMLO2*, are transcriptionally upregulated a few hours after infection with PM fungi [2, 11, 18].

Transcription is a very important biological process regulated at several stages. Understanding how gene regulation is orchestrated is an important challenge for characterizing complex events such as plant-pathogen interactions [19, 20]. Proteins known as transcription factors (TFs) modulate gene expression in specific ways through their binding to DNA regulatory elements [21]. Therefore, a key aim of gene expression analysis is the identification of transcription factors and DNA regulatory elements. Throughout the last decade, empirical data have accumulated suggesting that mutations in regulatory elements could be a major cause of phenotypic divergence [22, 23].

The term ‘promoter’ is used to designate the genomic sequence located upstream of a gene, which tends to contain cis-acting regulatory elements (CREs). Based on the distance from the transcription start site (TSS), the terms of ‘proximal promoter’ (several hundred nucleotides upstream of the TSS) and ‘distal promoter’ (thousands and more nucleotides upstream of the TSS) are also used. Most CREs are composed of 5–20 nucleotides and are localized in the proximal promoter [24]. It is generally thought that genes having similar expression patterns contain common motifs in their promoter regions [25].

The availability of whole genome data and next generation sequencing technology has opened up new avenues for the analysis of gene regulation and expression. The development of specialized databases of CREs in plants [26] and the development of bioinformatics tools to discover specific motifs in DNA sequences [27] greatly facilitate in silico analysis of promoters. Various methodologies can aid the identification of regulatory motifs, including deletion based functional analysis, comparative genomics, analysis of their co-expressed genes, and ChIP-Chip or ChIP-Seq [28–30] of co-expressed genes for overrepresented motifs [31, 32].

Prior to this study, a unique CRE was associated with the promoter region of the Arabidopsis susceptibility gene *AtMLO2* and co-expressed genes, which was found to be responsive to senescence, light stress, wounding and PM inoculation [33]. The aim of our research was to mine motifs and regulatory elements in the Putative Promoter Regions (PPRs) of *MLO* genes from different plant species, and to identify putative regulatory sequences specific for clade V homologs or for *MLO* genes known to be readily upregulated upon PM infection. The conclusions drawn by bioinformatics analysis were substantiated by the transcriptional characterization of *MLO* genes that we have recently described in the genome of melon [13].

## Results and discussion

### Identification and distribution of *MLO* CREs in Viridiplantae

Putative Promoter Regions (PPRs), corresponding to the 2 Kbp sequence located upstream the predicted TSS, were successfully extracted from 414 out of 447 *MLO* genes previously described in 25 plant genomes [13]. To identify CREs putatively involved in the transcriptional regulation of *MLO-*expression, a search was performed against the PLACE database. Three hundred and sixteen non-redundant CREs, distributed in variable number and order, were detected (Additional file 1: Table S1). In total, 186,060 elements were annotated in the PPRs of our 414 *MLO* gene dataset, and an average number of 449 CREs in forward- and reverse-complement orientation per homolog was detected (Table 1). *Capsicum annum* and *Arabidopsis thaliana* genomes had the highest (476) number of CREs per *MLO* homolog, whereas the *Brachypodium distachyon* genome had the lowest number (346)*.* The most abundant CREs were DOFCOREZM (PLACE-ID:S000265), CACTFTPPCA1 (ID:S000449) and CAATBOX1 (ID:S000028), which were recorded 10,983, 10,431 and 8533 times, respectively, in the whole dataset (Additional file 1: Table S1). DOFCOREZM is the core site required for binding of DOF (DNA-binding One zinc Finger) proteins, a family of transcription factors involved in many biological processes in higher plants [34]. The tetranucleotide CACT, present in the CACTFTPPCA1 element, is the key component of mesophyll expression in C4 plants [35, 36]*.*

**Table 1:** Candidate cis-regulatory elements (CREs) in the putative promoter regions (PPRs) of *MLO* genes

Family	Species	N. Analysed Promoters	N. CREs^a^	N. CREs/*MLO*
Volvocaceae	*Volvox carteri*	3 (3)^b^	1047	349
Chlamydomonadaceae	*Chlamydomonar reinhardtii*	4 (4)	941	235
Brassicaceae	*Arabidopsis thaliana*	15 (15)	7147	476
	*Brassica rapa*	23 (23)	10,212	444
	*Capsella rubella*	16 (16)	7288	456
Cucurbitaceae	*Citrullus lanatus*	14 (14)	6184	442
	*Cucumis melo*	15 (16)	6980	465
	*Cucumis sativus*	13 (13)	6105	470
Euphorbiaceae	*Manihot esculenta*	19 (19)	8899	468
Fabaceae	*Glycine max*	41 (41)	19,242	469
	*Medicago truncatula*	16 (16)	7170	448
	*Phaseolus vulgaris*	20 (20)	9418	471
Poaceae	*Brachypodium distachyon*	13 (13)	5157	397
	*Oryza sativa*	12 (12)	5282	440
	*Sorghum bicolor*	15 (15)	6145	410
	*Triticum aestivum*	26 (55)	10,595	408
Rosaceae	*Fragaria vesca*	17 (17)	7263	427
	*Malus domestica*	20 (21)	8533	427
	*Prunus persica*	19 (19)	8869	467
Solanaceae	*Solanum lycopersicum*	17 (17)	8051	474
	*Nicotiana tabacum*	14 (14)	6623	473
	*Capsicum annuum*	15 (15)	7139	476
	*Solanum melongena*	16 (18)	7489	416
	*Solanum tuberosum*	12 (12)	5573	464
Vitaceae	*Vitis vinifera*	19 (19)	8708	458
Total		**414 (447)**	**186,060**	**10,930**

^a^Referred to the cis-regulatory elements (CREs) annotated on the 2 Kbp upstream sequences from the putative initiation codon. ^b^In brackets, the number of total annotated *MLO* genes characterized by Iovieno et al. (2016)

**Fig. 1:**
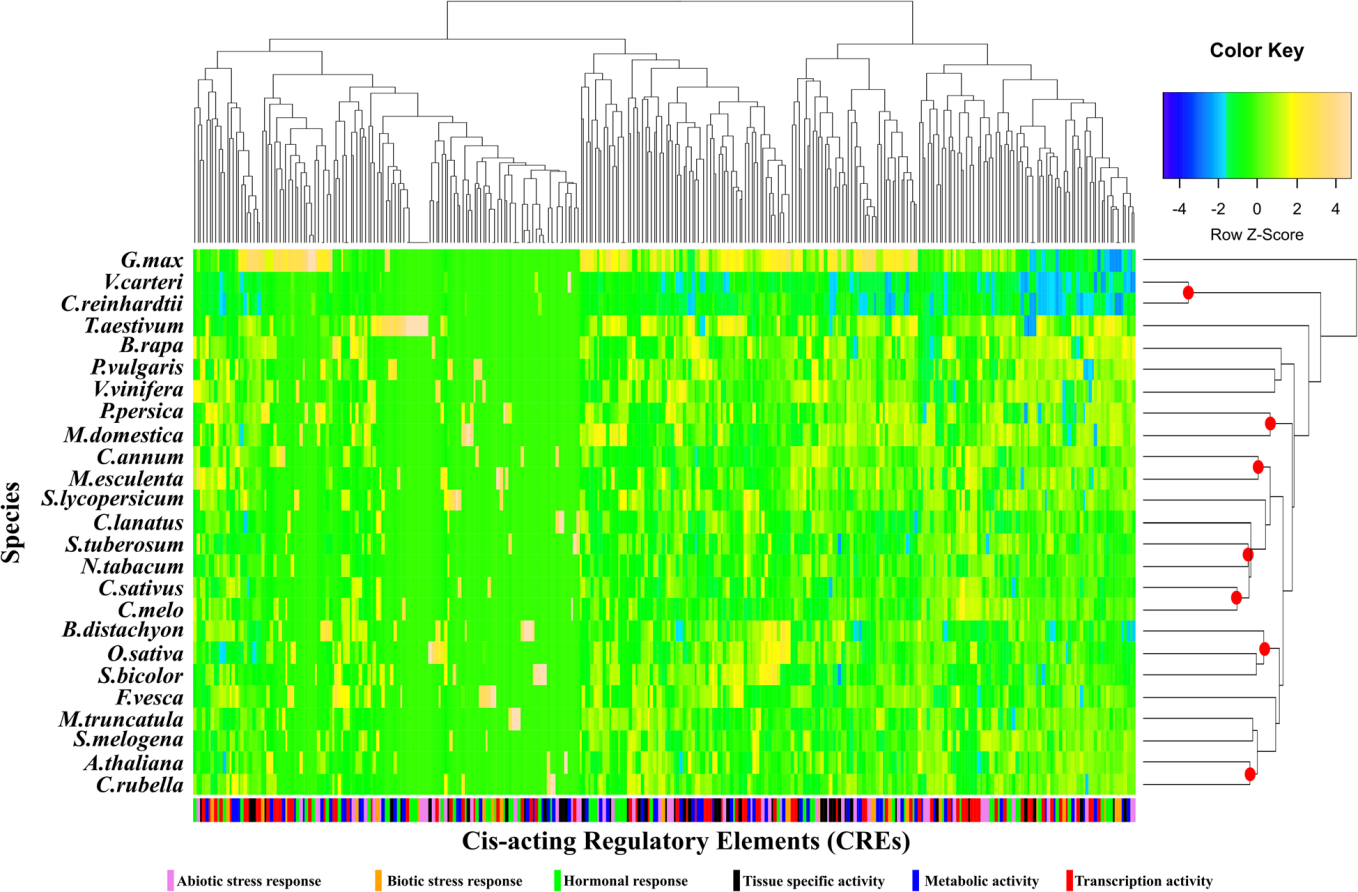
Graphical representation of the occurrence of 316 *MLO* cis-acting regulatory elements (CREs). The CRE dataset refers to the putative promoter region (PPR) of 414 *MLO* homologs occurring in 25 plant or algae species. The colour of heat map cells refer to the normalized Z-score, related to the standard deviation from the mean number of times a CRE occurs across species. Clustering on the right of the figure refers to species clustering according to the CRE profile, whereas clustering on the top of the figure refers to individual CREs. Red points indicate monophyletic-species groups. Information on the name and the functional category of individual CREs in provided at the bottom of the figure

Generally, flowering plants exhibited a family-specific pattern in terms of number of elements and global density distribution (Fig. 1). Indeed, homogenous CRE profiles were observed for Cucurbitaceae (*Cucumis melo* and *Cucumis sativus*), Rosaceae (*Malus domestica* and *Prunus persica*), Poaceae (*Brachypodium distachyon*, *Oryza sativa* and *Sorghum bicolor*), Solanaceae (*Solanum lycopersicum* and *Nicotiana tabacum*) and Brassicaceae (*Arabidopsis thaliana* and *Capsella rubella*). In other cases, the *MLO*-CRE profile appeared to be family-independent, such as in the case of *Brassica rapa* and *Glycine max*. This might be due to to missing information on the CRE sequence of some *MLO* family members, or the occurrence of true species-specific patterns. Analysis of individual CREs indicated that nineteen CREs were specifically present in monocots or eudicots. As expected, the *MLO* CRE profile of algae (*Volvox carteri* and *Chlamydomonar reinhardtii*) was clearly distinct from the one of flowering plants (Fig. 1), and included 147 elements which might be associated with conserved features during the evolution of green plants and involved in the transcription of *MLO* homologs.

### Distribution of *MLO* CREs in phylogenetic clades

**Fig. 2:**
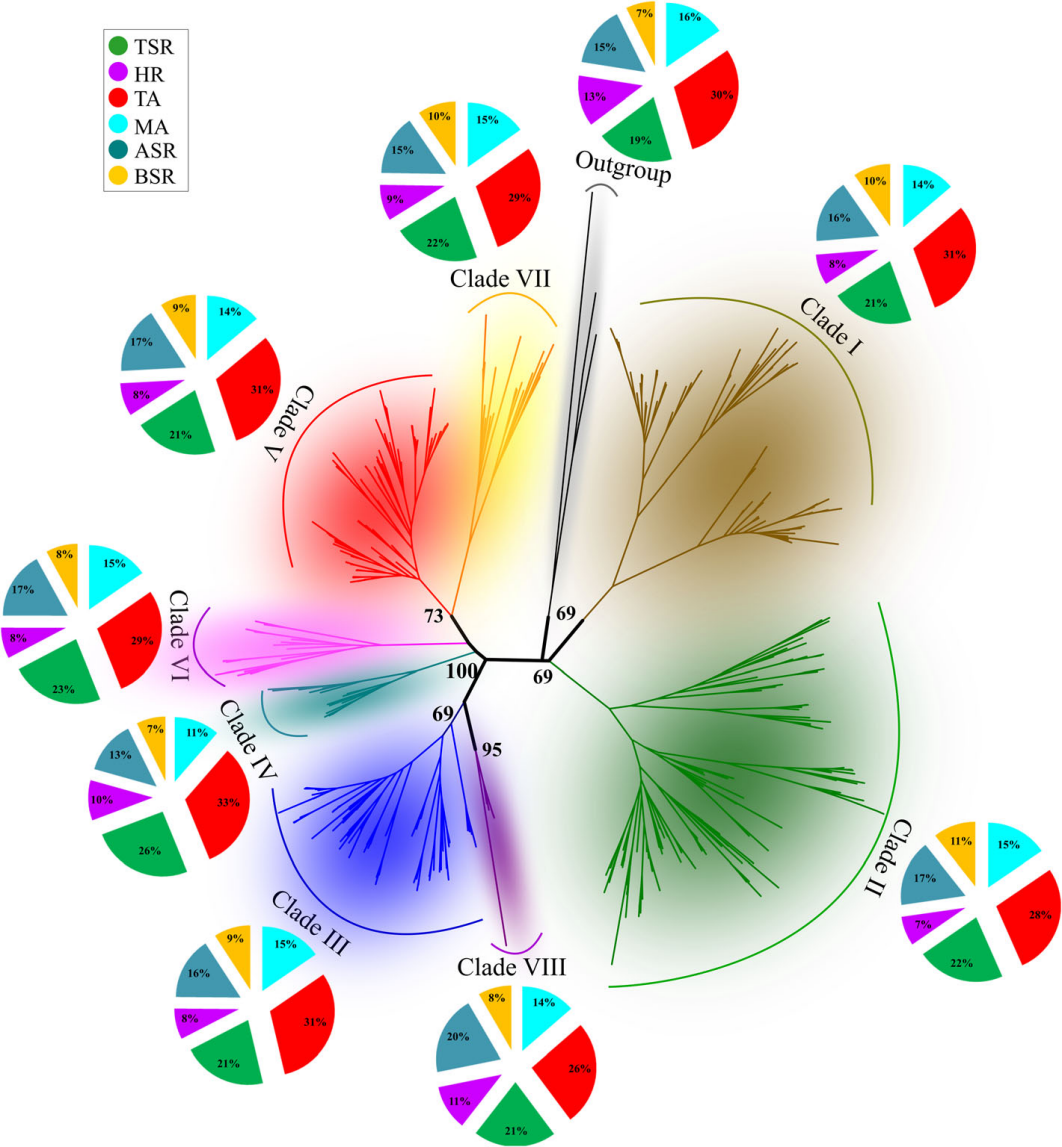
Correlation between the functional classification of CREs identified in this work and MLO proteins inference reported by Iovieno et al. (2016). The scheme depicts a phylogenetic tree of 396 MLO proteins from 25 genomes, indicating the distribution (%) of CRE identified in the *MLO*-PPRs of major clades (I-VIII) and outgroup (algae *MLOs*). The eight phylogenetic clades are indicated with different colours. The classification of CREs was based on the molecular function and the biological processes of the genes containing them. The 316 different CREs were grouped into 6 different classes: in blue “metabolic activity” (MA), in red “transcription activity” (TA), in green “tissue specific activity” (TSA), in violet “hormonal response” (HR), in cyan “abiotic stress response” (ASR) and in orange “biotic stress response” (BSR)

Identified CREs were subdivided in six functional categories: metabolic activity (MA) (63), transcription activity (TA) (74), tissue specific activity (TSA) (45), hormonal response (HR) (60), biotic stress response (BSR) (24) and abiotic stress response (ASR) (50). A phylogenic clade-specific CRE profile was obtained by normalizing, for each clade, the abundance of each CRE class on the total of CREs belonging to the same class **[CREs**^**CLADE CLASS**^**/CREs**^**CLASS**^**]** (Additional file 1: Table S2). Overall, the distribution of the classes was not markedly different among the eight phylogenetic clades under study (Fig. 2). MA, HR and BSR classes were evenly represented in all phylogenetic clades. A higher percentage of CREs associated with TA was observed in the *MLO*-PPRs of clade I, III and V (31%) and IV (33%). The highest percentage in CRE related to TSA and ASR was observed in the clades IV (26%) and VIII (20%) (Fig. 2). A core of 119 (~ 40% of total) CRE elements was highly conserved in all regulatory profiles of the eight clades. This subset could help to explain the coordinate upregulation of *MLO* homologs from different clades in response to the same biotic or abiotic stress [37]. Possibly, a core regulatory structure of PPRs of *MLO*s belonging to different clades promotes a basic gene activation to environmental cues. No specific CRE was identified in either pathogen-responsive or clade V *MLO* genes (Table 2).

**Table 2:** Features of *MLO* genes upregulated upon powdery mildew infection and/or belonging to the phylogenetic clade V

Species	Upregulated	Clade V	Phylogenetic clade (Iovieno et al., 2016)	Consortium gene ID	Reference for upregulated *MLOs*
*Arabidopsis thaliana*	AtMLO2	AtMLO2	V	AT1G11310	[48]
	AtMLO3	–	VI	AT3G45290	
	AtMLO6	AtMLO6	V	AT1G61560	
	AtMLO12	AtMLO12	V	AT2G39200	
*Vitis vinifera*	VvMLO3	VvMLO3	V	GSVIVG01025653001	[13, 49]
	VvMLO4	VvMLO4	V	GSVIVG01025162001	
	VvMLO9		VII	GSVIVG01020617001	
	–	VvMLO13	V	GSVIVG01016302001	
	VvMLO17	VvMLO17	V	GSVIVG01016304001	
*Malus domestica*	MdMLO11	MdMLO11	V	MDP0000239643	[50]
	–	MdMLO5	V	MDP0000163089	
	–	MdMLO7	V	MDP0000123907	
	MdMLO18	–	VII	MDP0000928368	
	MdMLO19	MdMLO19	V	MDP0000168714	
*Capsicum annuum*	–	CaMLO1	V	Capana11g000102	[10]
	CaMLO2	CaMLO2	V	Capana06g001935	
*Solanum lycopersicum*	SlMLO1	SlMLO1	V	Solyc04g049090	[17]
	–	SlMLO3	V	Solyc06g010030	
	SlMLO4	–	III	Solyc00g007200	
	–	SlMLO5	V	Solyc03g095650	
	–	SlMLO8	V	Solyc11g069220	
	SlMLO14	–	I	Solyc07g063260	
*Cucumis sativus*	CsMLO1	CsMLO1	V	Csa1M085890.	[51]
	–	CsMLO8	V	Csa5M623470	
	–	CsMLO11	V	Csa6M292430	
*Citrullus lanatus*	–	ClMLO2	V	Cla005044	[7, 13]
	–	ClMLO5	V	Cla008753	
	ClMLO12	ClMLO12	V	Cla020573	
*Cucumis melo*	–	CmMLO3	V	MELO3C005044	This work
	CmMLO5	CmMLO5	V	MELO3C012438	
	–	CmMLO12	V	MELO3C025761	

In order to identify CREs enriched in specific phylogenetic clades, a motif enrichment analysis was performed. Five clades (I, II, III, IV and V) displayed significantly enriched CREs (e-CREs) (Additional file 1: Table S3). In total, 43 over-represented CREs were identified, of which about 40% were grouped into the TA-class (Additional file 1: Table S3). *MLO* PPRs clustering in clades I and II displayed enrichment of specific transcription factor binding sites (MYCATRD22 for clade I; RAV1BAT and WBOXNTCHN48 for clade II). Clade III showed five e-CREs (POLASIG1, POLASIG2, GT1GMSCAM4, TATABOX5 and MARTBOX). The element AGCBOXNPGLB, associated with stress signal-response factors (as ER and PR-proteins), was the only CRE significantly enriched in clade IV. Interestingly, more than 50% (24 out of 43) of identified e-CREs, mostly related to the TA class, were associated with PPRs of *MLO* homologs clustering in clade V (adjusted *p*-value < 0.01).

### De novo identification of putative regulatory motifs in pathogen-responsive *MLOs*

**Fig. 3:**
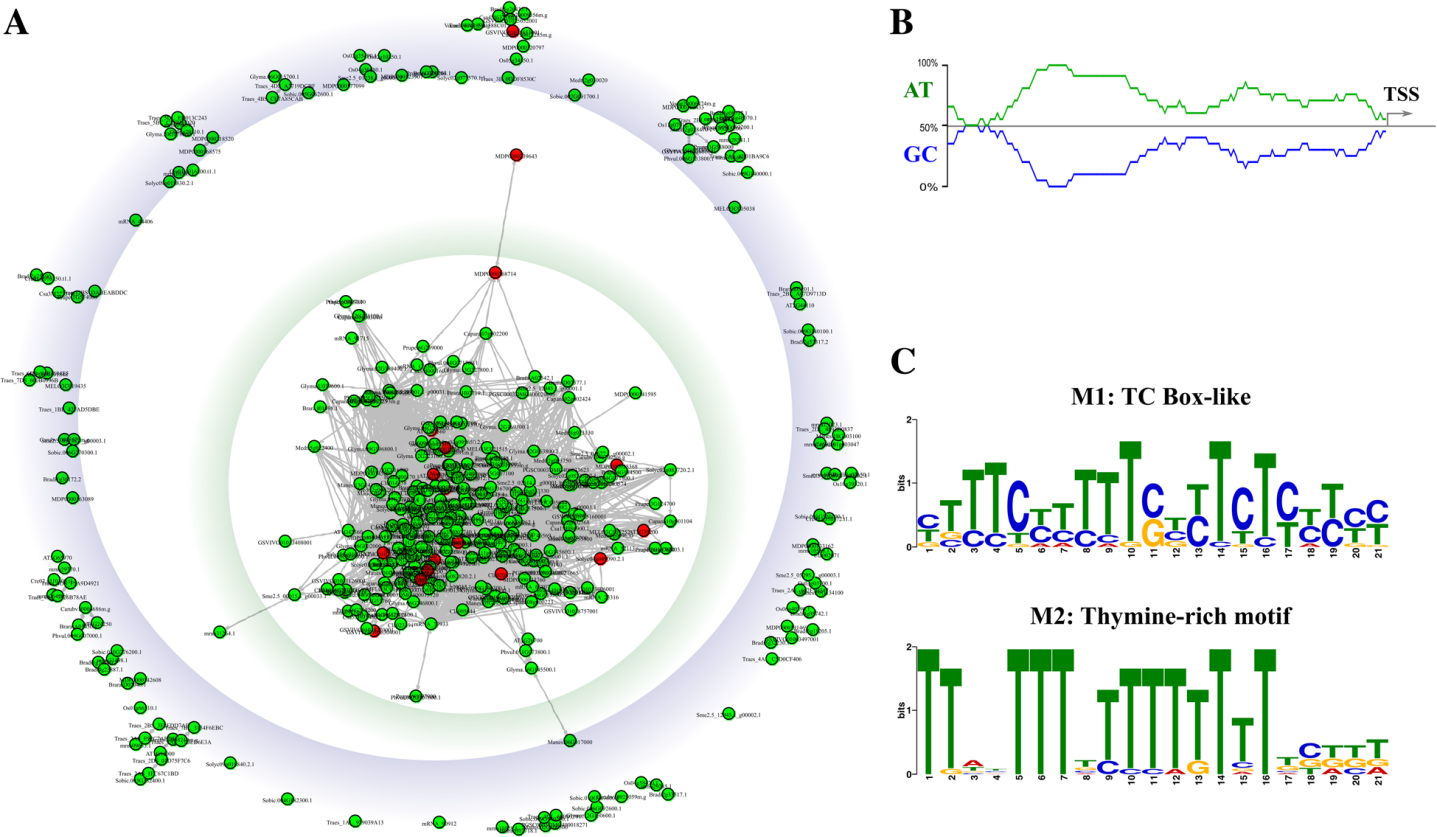
Network analysis of putative promoter sequences and novel motifs discovered in the putative promoter regions (PPRs) of upregulated *MLO* genes. **a**) Sequences similarity network of 414 *MLOs* based on MAFFT similarity matrix of 200-bp 5′ upstream from the translation start site. Circles (nodes) show the PPRs (red for upregulated *MLO*s; green for remaining homologs) and lines (edges) indicate the connections. The sequence identity cut-off, used to generate the network edges, is > 30%. **b**) Typical distribution of PPR AT-content *MLO* genes upregulated upon PM challenge. **c**) Sequence logo of two motifs (M1 and M2) identified in the PPRs of *MLO* upregulated upon PM challenge. M1: Thymine-rich motif; M2: TC box-like

Additional regulatory motifs in PPRs of transcriptionally responsive *MLO*-homologs were disclosed. From a first global alignment of the PPR-dataset, the highest sequence identity was observed in the first 200 bp upstream of the predicted TSS. A similarity matrix restricted to this region highlighted the occurrence of a large cluster containing about 65% of the PPRs, including those of 17 (out of 18) *MLO* genes known to be upregulated upon PM infection (Table 2 and Fig. 3a). Multiple alignment of the same region revealed higher sequence identity (33%) and AT-content (70%) in the PPR of *MLO* homologs upregulated upon PM infection compared to the rest of the dataset (27 and 64%, respectively) (Fig. 3a and b). Interestingly, a significant association between expression and the AT-content of PPRs was previously reported for genes different from *MLOs* [38].

A MEME D-mode analysis was carried out to identify enriched motifs in the PPR of *MLO* genes upregulated during the interaction with PM fungi or included in clade V (Table 2). Notably, two overrepresented motifs (M1 and M2) were found in *MLO* genes up-regulated upon PM infection (Additional file 1: Table S4). Except for *AtMLO3*, these motifs were present within the 0.4 Kbp sequence upstream the predicted TSS. Three conserved motifs (M3, M4 and M5) were over-represented in clade V homologs (Additional file 1: Table S4). The consensus sequence of M1 (Fig. 3c) is similar to a TC-element sequence. Previous studies suggested that TC-elements might constitute a novel class of regulatory sequences participating to the complex modulation of expression of plant genes expressed in specific conditions [39]. The highly conserved (~ 70% identity) M2 motif is characterised by a high content of thymine residues. The thymine-rich motif was reported as an essential element for transcription efficiency, since it may serve to increase the accessibility of downstream promoter sequences for additional protein factors [40, 41]. The high variability of the remaining M3, M4 and M5 sequences in the PPR-dataset complicates the prediction of their putative role.

Based on Genome Ontology (GO) association analysis, the motifs from M1 to M5 were predicted to be associated with trans-membrane transport (GO:0005886 and GO:0006813), positive regulation of transcription (GO:0016563) and transcription factor activity (GO:0045449, GO:0003700 and GO:0045941). The motif M2 is associated with the GO:0001950 term of *S. cerevisiae* promoter regions of genes encoding proteins of plasma membrane enriched fraction. Overall, these results are in accordance with the transmembrane localization of MLO proteins and their supposed role in transmembrane transport [14].

The PPRs of 414 *MLO* genes were also analysed by MAST to verify the occurrence of the CRE motif previously characterized by Humphry et al. (2010) [33] in the Arabidopsis susceptibility gene *AtMLO2* and co-expressed genes. Remarkably, this motif was found in about 96% (408) of *MLO* promoters. Experiments evaluating the expression pattern of the β-glucuronidase (GUS) reporter gene driven by this motif highlighted strong signal in several organs and in response not only to PM inoculation, but also to senescence, light stress and wounding [33]. Together, these results suggest that this motif might represent a general signature of *MLO* genes, regulating expression in response to homeostasis perturbations.

### Transcriptional validation of bioinformatics results

**Fig. 4:**
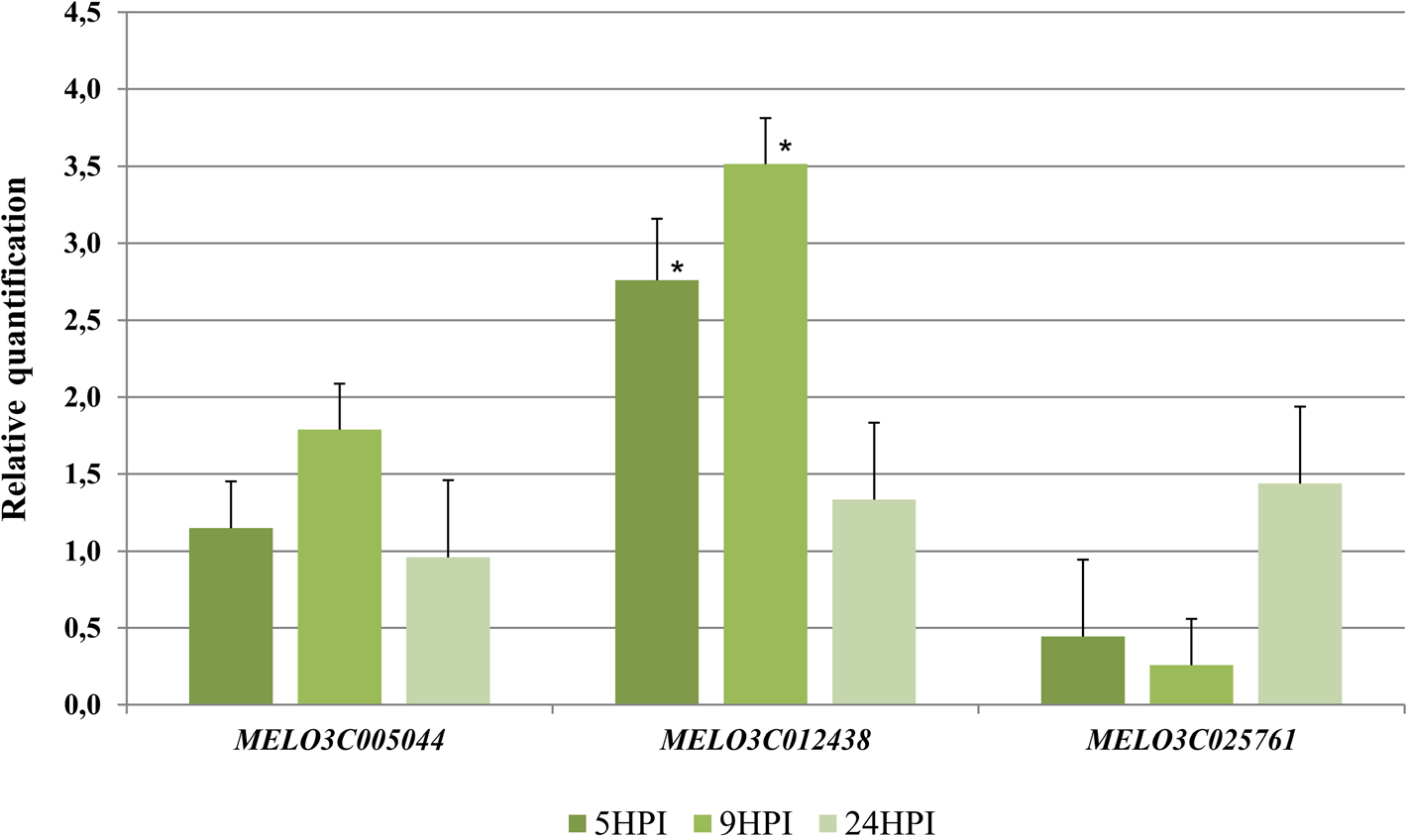
Relative abundance of *Cucumis melo* clade V *MLO* gene transcripts in response to PM challenge compared to mock-treated plants. Data refer to three different time points, at 5, 9 and 24 h post inoculation (HPI) with the PM fungus *Podosphaera xanthii*. Standard error bars refer to three biological replicates. Significant differences among the means were inferred using the Student’s t test for each time point. Asterisks indicate *p* < 0.05

In silico analyses highlighted the overrepresentation of two putative PPR regulatory elements, namely a TC box-like and a thymine-rich motif, in *MLO* homologs up-regulated upon PM infection. As proof of concept, we analysed the transcriptional response of three clade V genes previously found in the genome of melon [13] (*MELO3C005044: CmMLO3*, *MELO3C012438: CmMLO5* and *MELO3C025761: CmMLO12*). Among them, *MELO3C012438* shows the best combined match (combined *p*-value <1e-5) for the over-represented motifs in the PPR region. Following artificial inoculation with the PM fungus *Podosphaera xanthii*, a strong upregulation of *MELO3C012438* was observed at both 5 and 9 h post-inoculation. In contrast, no significant difference was detected for *MELO3C005044*, and *MELO3C025761* (Fig. 4). As eudicot *MLO* susceptibility genes belong to clade V and are upregulated upon PM challenge [2, 11, 18], our results indicate that *MELO3C012438* is a PM susceptibility factor in melon.

## Conclusions

Mechanisms controlling the expression of *MLO* homologs are not clear. Here we report the characterization of *MLO* PPRs in a large dataset extracted from 25 plant and algal species. Distribution patterns of putative *MLO* regulatory elements varied according to genomic relatedness among species and phylogenetic distance among homologs. Two motifs, referred to as M1 and M2, were overrepresented in the PPRs of clade V *MLO* genes upregulated upon PM infection, which are known to act as PM susceptibility genes. In accordance with this finding, we showed that, in melon, a clade V *MLO* gene enriched for M1 and M2 is upregulated following challenge with the melon PM fungus *Podosphaera xanthii*. Overall, this study provides insight on the PPR of the *MLO* gene family, reports the characterization of a candidate *MLO* susceptibility gene in melon, and highlights PPR diagnostic features that could be used to identify *MLO* susceptibility genes across cultivated species. This information is extremely valuable for breeding purposes, as the inactivation of *MLO* susceptibility genes, for example by targeted silencing and genome editing, might lead to the identification of PM resistant genotypes.

## Methods

### Putative promoter region dataset

447 *MLO* genes from twenty-five different genomes (*Volvox carteri*, *Chlamydomonas reinhardtii*, *Arabidopsis thaliana*, *Brassica rapa*, *Capsella rubella*, *Citrullus lanatus*, *Cucumis melo*, *Cucumis. sativus*, *Manihot esculenta*, *Glycine max*, *Medicago truncatula*, *Phaseolus vulgaris*, *Brachypodium distachyon*, *Oryza sativa*, *Sorghum bicolor*, *Triticum aestivum*, *Fragaria vesca*, *Malus domestica*, *Prunus persica*, *Solanum lycopersicum*, *Nicotiana tabacum*, *Capsicum annuum*, *Solanum melongena*, *Solanum tuberosum* and *Vitis vinifera*), described in the work of Iovieno et al. (2016), were considered as initial dataset. Putative promoter regions (PPRs) (2 Kbp upstream the predicted TSS reported in gene annotations) were extracted using BEDTools v2.17.0 (http://samtools.sourceforge.net) and 414 PPRs (out of 447) with a PPR length > 1 Kbp were used further.

### Detection and distribution of CREs in Viridiplantae *MLO* homologs

PPR-sequences were scanned in forward and reverse complement orientation to search for cis-acting regulatory elements (CREs) present in the Plant cis-acting regulatory DNA elements (PLACE) database (https://sogo.dna.affrc.go.jp/cgi-bin/sogo.cgi?lang=en&pj=640&action=page&page=newplace) [26]. The most stringent identification criterion (exact match) was used. The Needleman-Wunsch algorithm (default setting) was used to generate a non-redundant dataset by eliminating duplicated motifs defined by ‘identical sequences’. In order to obtain a CRE map of Viridiplantae *MLO* homologs, a hierarchical clustering analysis of annotated CREs was performed. A heat map was generated starting from a CRE-abundance matrix using the ‘GPLOTS’ R software package [41].

### Characterization of CREs with respect to phylogenetic clades and transcriptional response

Identified CREs (316) were manually assigned to six different functional categories (classes) based on the regulatory functions reported in literature for each element:

- **Metabolic activity** (MA): implicated into biosynthesis of primary and secondary metabolites;
- **Transcription activity** (TA): recognised by transcription factors;
- **Tissue specific activity** (TSA): associated to a specific tissue;
- **Hormonal response** (HR): induced by phytohormones;
- **Biotic stress response** (BSR): induced by biotic stresses;
- **Abiotic stress response** (ASR): induced by abiotic stresses.

A clade-specific CRE profile was obtained by normalizing, for each clade, the abundance of each CRE class on the total of CREs belonging to the same class **[CREs**^**CLADE CLASS**^**/ CREs**^**CLASS**^**]**. In order to identify CREs overrepresented in specific clades, PPRs associated with each clade were compared to those of the remaining dataset, using the Analysis of Motif Enrichment (AME) tool available in the MEME suite and the default cut-off value [42]. Motif enrichment significance was assessed using the Ranksum test, also known as the Mann-Whitney U test, with subsequent multiple testing correction applied to the obtained *P* values (adjusted *p*-value < 0.05). Analysis of motif enrichment and individual CRE annotation were also carried out to compare PPR of homologs upregulated during PM infection or included in clade V with those of other *MLO* homologs.

### PPR similarity network

MAFFT version v6.814b was used to align the first 200 bp of PPRs, and to generate a similarity matrix, using the EINS-i algorithm [43]. A weighted correlation network analysis was carried out using the ‘WGCNA’ R software package [44].

### De novo annotation and function prediction of *MLO*-motifs

The Multiple EM for Motif Elicitation (MEME) algorithm [27] was used to identify motifs that are specifically enriched in the PPRs of *MLO* genes included in clade V or previously shown to be upregulated upon PM challenge (Table 2). The analysis was carried out in discriminative-mode (D-mode), using the default cut-off value for statistical confidence.

The best combined match of enriched motifs identified by MEME analysis was detected using Motif Alignment & Search Tool (MAST) (default cut-off: p-value 1e-4 and E-value < 10) [27]. MAST was also used to confirm the presence of the motif previously described by Humphry et al. (2010) [33] in the PPR dataset, using two different settings (p-value <1e-4 and p-value <1e-3). Enriched motifs were analysed using the Gene Ontology for Motifs (GOMo, http://meme-suite.org) tool [29] to determine if any motif was significantly associated with genes linked to one or more Genome Ontology (GO) terms [45].

### Transcriptional characterization of melon *MLO* genes

Plants of *Cucumis melo* (cv. Tendral Verde TS258) were inoculated with the PM fungus *Podosphaera xantii* by brushing heavily infected leaves collected in the field. Plants brushed with healthy leaves were used as experimental controls. Leaf samples were collected at 5, 9 and 24 h after inoculation (three biological replicates for each treatment and three technical replicates). Protocols for RNA isolation, cDNA preparation and qRT-PCR were the same as those reported by Iovieno et al. (2015) [7]. Relative transcript abundance of the three clade V melon genes *MELO3C005044*, *MELO3C012438* and *MELO3C025761* was assessed using the 2^−ΔΔCt^ method [46], using the β-actin gene as housekeeping for target gene normalization [47]. The Student’s t-test was used to test the null hypothesis. All the primer pairs used for gene expression analysis are listed in Additional file 1: Table S5. For each primer pair, a dissociation kinetics analysis was performed to ensure the specificity of the amplification product and amplification efficiency was assessed by generating a standard curve with five serial 10-fold dilutions.

## Additional file

Additional file 1: **Table S1.** List of 316 candidate CREs in the putative promoter regions of MLOs characterized by Iovieno et al., (2016). **Table S2.** Functional annotation of CREs in *MLO*-PPRs. The classification is based on the molecular function and the biological processes of the genes containing CREs. **Table S3.** List of enriched-CREs identified for each phylogenetic clade. **Table S4.** List of enriched motifs identified in the putative promoter regions (PPRs) of *MLO* genes upregulated during the interaction with PM fungi and/or belonging to the phylogenetic clade V. **Table S5.** Gene-specific primers used in this study for gene-expression analysis and their amplification efficiency. (XLSX 31 kb)

## Abbreviations

AME: Analysis of Motif Enrichment
ASR: Abiotic Stress Response
BSR: Biotic Stress Response
CRE: Cis-acting Regulatory Element
e-CREs: Enriched-CRE
GO: Genome Ontology
GOMo: Gene Ontology for Motif
HR: Hormonal Response
MA: Metabolic Activity
MAST: Motif Alignment & Search Tool
MEME D-mode: Multiple EM for Motif Elicitation Discriminative-mode
MLO: Mildew Locus O
PLACE: Plant cis-acting regulatory DNA element
PM: Powdery Mildew
PPR: Putative Promoter Region
TA: Transcription Activity
TSA: Tissue Specific Activity
TSS: Transcription Start Site
